# Health effect of public sports services and public health services: empirical evidence from China

**DOI:** 10.3389/fpubh.2024.1320216

**Published:** 2024-05-13

**Authors:** Lin Cao, Jianguang Cai, Yanping Gong, Qingqing Bao, Junrong Hu, Ningxiao Tang

**Affiliations:** ^1^School of Physical Education, Hunan University of Science and Technology, Xiangtan, China; ^2^Business School, Guilin University of Electronic Technology, Guilin, China; ^3^Outdoor Sports Academy, Guilin Tourism University, Guilin, China; ^4^Department of Sports, Guilin University of Electronic Technology, Guilin, China

**Keywords:** public sports services, public health services, health effect, integration of sports and medicine, national health

## Abstract

There is no clear explanation for the extraordinary rebound in China’s population mortality over the past decade. This paper utilizes panel data from 31 Chinese provinces from 2010 to 2020 to determine the distinct impacts of public sports services (PSS), public health services (PMS), and their interaction on population mortality. Empirical results show that public sports services significantly reduce mortality. Every unit increase in public sports services reduces mortality by about 2.3%. It is characterized by delayed realization. Public health services were surprisingly associated with a rebound in mortality. Further studies found strong health effect from interaction of public sports and health services. The effect was significantly strengthened in areas with fewer extreme temperatures or developed economy. The findings have important policy implications for the high-quality development of public sports and health services. It also emphasizes integration of sports and medicine and mitigates health risks associated with extreme temperatures.

## 1 Introduction

National Health is a greater priority today than ever before in the national development agenda. It serves as the cornerstone for a nation. The Chinese Government has traditionally attached great importance to the nation’s health and well-being. The average life expectancy in 2021 for China is 78.2, higher than that of middle-and high-income countries (1). The health indicators of infant mortality and maternal mortality show a clear downward trend in the period 1949–2021. The mortality rate of the population in China has experienced an increase from 6.4 to 7.2% between the years 2000 and 2021. This statistic is widely acknowledged by international organizations such as the World Bank and the World Health Organization as a reliable measure of health status (2). Obviously, this is unpopular but objectively true. As far as we know, there is no clear explanation for the abnormal phenomenon of mortality rebound in China in the past 10 years.

National health is affected by many factors. In the view of Patwardhan, Mutalik (3), health is both an active process of individual participation and a passive process of disease treatment. Public sports services and public health services are therefore two fundamental determinants of national health. As a cross-border issue, health must be considered from the perspective of public services. In terms of the positive impacts on public health, it is worth noting that both ancient Chinese philosophy and contemporary scientific research hold that public sports services play a crucial role in enhancing overall well-being (4, 5). At present, sports medicine has developed into a mature discipline, which mainly studies the relationship between exercise and health and its mechanism from the individual micro level (6–8). A lot of theoretical literature has concluded that health satisfaction is most closely linked to national satisfaction with physical exercise participation and that public sports services are necessary to improve national health (9, 10). However, it is a pity that compared with the research in the field of sports medicine, most of the literature on the health effect of public sports services is based on empirical judgment or theoretical interpretation, and no quantitative research literature has been found. Furthermore, public health services constitute a significant determinant of national health. As an important system implemented by the government, its effect has been concerned by many scholars. Much of the literature argues that whether or not public health service improves national health depends largely on the level of economic development (11). Primary health care can play a great role in reducing population mortality when a country is at a low level of economic development. A case in point is the rural health workers in China in the last century. They have greatly alleviated the mortality of accidental injuries in remote and rural areas, and greatly reduced the mortality of newborns, pregnant women, and children in rural areas (12). Nevertheless, when the level of economic development is significantly improved, does the marginal health effect of public health services exist? Many documents have come to unexpected conclusions. Filmer and Pritchett (13) argue that spending money on healthcare does not always translate into better health, because there is a transmission mechanism of “public health services-efficiency of government supply-actual utilization of residents-national health “. A variety of factors, such as healthcare sector administrative performance, commercialized supply of health services, and medical science and technology, have complex impacts on health effect of public health services. Filmer et al. (14) revealed disappointing experiences in the implementation of primary healthcare programs in developing countries. Jingyuan et al. (15) found that healthcare costs are increasing at a rate of nearly 20% per year, but population mortality has remained high. The same finding was reached by Jiangang and Yuxi (16), who observed that China’s death index has shown a relatively fast downward trend since 1997, but there was a significant turnaround with a rebound in the death rate in 2017.

This raises the following two questions: How do public sports services specifically affect national health in China? Is investment in public health services effective for national health or not? Although existing literature explores the relationship between public sports and health services and national health, it does not reveal the rebound in population mortality over the last 10 years.

To this end, this paper incorporates both public sports services and public health services into the research framework. By employing inter-provincial panel data encompassing 31 provinces in China over the period spanning from 2010 to 2020, the study endeavors to assess the effects of these services on population mortality.

Compared with the existing literature, the marginal contributions of this paper mainly include: first, the health effect of public sports services has lagged and delayed gratification. This not only provides new information for sports economics and health economics but also provides a theoretical basis for a sustainable supply of public sports services. Second, we incorporate both public sports services and public health services into our research model, which differs from the majority of literature that focuses solely on whether public health services significantly improve health (17, 18). The strong health effects of interaction between the two are revealed. Merely augmenting public health services does not yield advantageous outcomes for national health, which sheds new light on the importance of integration of sports and medicine. Third, heterogeneity analysis shows that the health effect of interaction between public sports and health services are weakened in areas economically disadvantaged, but were significantly enhanced in areas where extreme temperatures were less frequent. It provides new insights into health imbalances across regions in China.

The remaining structure of this paper is as follows: the second part puts forward the research hypothesis based on review of literature related to public sports services, public health services, and national health. The third part is the data source, variable definition, and the research model. The fourth part is baseline regression and robustness checks. The fifth part discusses health effect of interaction of public sports and health services and heterogeneity analysis based on economic development and extreme temperatures. The sixth part is research conclusions, policy implications, and limitations.

## 2 Literature review and research hypothesis

### 2.1 Public sports services and national health

At present, the spectrum of human diseases has shifted from acute or severe infectious diseases to chronic diseases, becoming the “first killer” of health. According to the World Health Organization (WHO), current threats to national health come mainly from malignant tumors, cardiovascular diseases, diabetes, and hypertension. Studies have confirmed that the above diseases are mainly due to insufficient physical activity (19). Physical activity has been well documented for physical and mental health (20, 21). Therefore, it is widely regarded as a panacea for health (22). The World Health Organization (WHO) regards physical activity (PA) as the primary consideration in the global health field, proposing a standard at least 150 min of moderate-intensity physical activity throughout the week (23). However, available data suggests that a quarter of adults globally do not meet this standard, and health risks associated with physical inactivity further exacerbate the challenge (24). Physical inactivity is the fourth leading risk factor for death globally, after hypertension, smoking, and hyperglycemia (25). Healthy life is closely related to perfect public sports services (26). Therefore, expanding access to public sports programs is a crucial step in the advancement of national health.

China, as an emerging global economic force, has experienced consistent and rapid economic expansion for over four decades. This continuous prosperity has laid a robust material groundwork for the positive impact of public sports services on national health. According to the China Sports Statistics Yearbook, the allocation of funds for public sports services constituted 10.43% of the total sports expenditure in 2021. The indicator reaches a maximum value of merely 0.8% in Beijing, while the province with the lowest value is Heilongjiang, with a mere 0.06%. As a result, China’s public sports services still suffer from insufficient total investment and regional imbalances, which inevitably affect national health.

However, given the comparatively low availability of public sports services in China, we can apply the marginal theory in public economics to conclude that the current investment in public sports services exhibits a growing marginal effect. This means that increasing public sports services will help to realize its health promotion effect.

Therefore, the first research hypothesis of this paper is proposed:

H1: With the increase in public sports services, China's national health will be significantly improved.

### 2.2 Public health services and national health

There is still no consensus on the health outcomes of public health services. There are currently opposing views as follows:

First, the theory of “insignificant relationship.” The early research generally argues that public health services have little or no impact on health status. Auster et al. (27) found that the age-adjusted death rate has not declined appreciably from 1955 to 1965, in spite of a substantial increase in the quantity of medical services produced per capita. St Leger et al. (28) obtained the same results that there was no negative correlation between health-service factors and deaths in 18 developed countries. Newhouse and Friedlander (29) examined 1959–1962 US Health Examination Survey data and found that additional healthcare resources have little effect and that individual actions may be more important to health than local medical resources.

Second, the theory of “health improvement.” In recent years, research has found health expenditure is at least a significant explanatory variable for certain health indicators in some countries (30). Cutler et al. (31) famously argued that modern healthcare has great benefits for quality of life. A study conducted on OECD countries revealed that more spending on pharmaceuticals is associated with an increase in life expectancy among the population with a marginal effect that appeared to diminish with age (32). But, there is a limited impact of increased pharmaceutical expenditure on the amelioration of severe illnesses like cancer (33).

There are very few studies on a possible positive correlation between public health services and mortality. Cochrane et al.(34) found that there is a marked positive association between the prevalence of doctors and mortality in the younger age groups. Clearly, the alarming relationship deserves serious consideration. However, no more reasonable explanation has been found for this abnormal phenomenon at present.

Why might there be a strong positive correlation between public health services and mortality in certain age groups or countries? This paper argues that a preliminary explanation may be related to environmental pollution, and its damage to health can not be ignored. Morbidity and mortality associated with environmental pollution continue to increase until this point (35, 36). Based on a publication in The Lancet, it was shown that ambient PM2.5 emerged as the fifth most significant risk factor for mortality. In the year 2015, exposure to PM2.5 was responsible for causing around 4.2 million deaths, which accounted for approximately 7.6% of the overall global mortality rate (37). The morbidity and mortality linked to air pollution are quite high in China, reaching as high as 40% in the city of Beijing (38). The study on disease burden in China has shown that air pollution is the fourth health risk factor in China. Thus, deterioration of mortality caused by environmental pollution increases demand for public health services, resulting in a strong positive correlation between public health services and population mortality. The correlation may not be significant in developed countries where environmental quality is generally better. China has inevitably caused environmental pollution in the process of rapid economic development, significantly increasing the burden on the healthcare sector. It is important to note that provision of public health services cannot be considered the main trigger for deterioration of mortality.

Therefore, the second research hypothesis of this paper is proposed:

H2: There is a significant positive correlation between public health services and population mortality in China.

### 2.3 Interaction between public sports and health services

There are two different perspectives on the specific pathways of public health services to national health.

First, the argument of “direct effect”. “Treating diseases and saving lives” is the early understanding of the function of public health services. However, the view overemphasizes passive health concepts centered on “treating diseases” and tends to lead to an over-reliance on public health services. As mentioned earlier, most of the literature confirms that public health services do not have a clear direct relationship with national health, or that its direct impact is weak. The report of the World Health Organization confirms that 40% of the problem of human healthy life span lies in genetics and objective environmental conditions: of which 15% are genetic factors, 10% are social factors, 7% are living environment and geographic climate conditions, 60% are good healthy lifestyle, and 8% are medical conditions (39). Obviously, not much is expected from the medical factor. In addition, the weak direct effect of public health services on health may be further weakened or even reversed by a range of factors, including management efficiency of the public sector, crowding out effects from the market, and so on (14).

Second, the argument of “integration of sports and medicine.” The core logic of the theory is “active health, prevention-oriented, integrated prevention and treatment,” and the core idea is “exercise is medicine.” It emphasizes both the primary role of physical activity in health promotion and complementary strength and deep integration between sports and healthcare, which are extremely important for health and well-being of human beings (40).

The health benefits of sports and healthcare might be significantly reduced if the two are artificially separated. Modern medical science provides a rich theoretical basis for health promotion in sports. Among them, the primary focus of preventive medicine is to research individuals who are in excellent health and those who do not exhibit symptoms of illness. This approach aligns closely with contemporary health concepts that emphasize proactive health practices, active health, and self-management of life. Interestingly, the Exercise is Medicine initiative is co-sponsored by the American College of Sports Medicine (ACSM) and the American Medical Association (AMA). The health impact of public sports services would be significantly diminished in the absence of effective collaboration with robust public health services. These two components exhibit a mutually reinforcing relationship, as they, respectively, address the preventive and curative aspects of disease management. It is beneficial for human health and well-being to enhance integration of sports and medicine and adopt the health management approach that addresses both the symptoms and the root causes of health problems.

Therefore, the third research hypothesis of this paper is proposed:

H3: The interaction between public sport and health services has a strong improvement in health.

## 3 Methodology

### 3.1 Data sources

The study data is from China Health Statistics Yearbook, China Sports Statistics Yearbook, China Statistical Yearbook, China Education Statistics Yearbook, and China Environmental Statistics Yearbook. Balanced panel data for 31 provinces, autonomous regions, and municipalities in China from 2010 to 2020 are finally constructed. The caliber of statistical data and indicators in Hong Kong, Taiwan, and Macao are not consistent, so they are not covered in this paper.

### 3.2 Variable description

#### 3.2.1 Dependent variable

##### 3.2.1.1 National health (MOR)

This paper uses population mortality to measure national health for the following reasons: first, in the report “Investing in Health” of the World Bank, mortality is widely used to investigate differences in residents’ health within a country or region (2). Second, the general absence or relative instability of data such as morbidity and life expectancy does not allow for a comprehensive and accurate measurement of national health. Third, the comprehensive health indicators based on first-hand survey data still suffer from limited sample size and underrepresentation. Finally, indicators such as perinatal, maternal, and infectious disease mortality rates hardly reflect the overall health status of all populations. Therefore, we use population mortality (MOR) as the dependent variable.

#### 3.2.2 Independent variable

##### 3.2.2.1 Public sports services

Drawing on the approaches of Fengbiao and Jiahong (41), the indicator system for public sports services is constructed from three dimensions including financial input, sports venues, and staff organization. Among them, financial input includes the proportion of expenditure on sports to local financial expenditure, per capita expenditure on sports, and per capita financial allocation for sports; Sports venue consists of the total number of sports venues per 10,000 people and the per capita area of public sports venues. The staff input includes the number of social sports instructors per 10,000 people, the number of sports administrative organs per 10,000 people, and the employees of administrative organs per 10,000 people. The entropy method was used to determine the weights of these three indicators, then summing products of weights and indicators to calculate the total score of public sports services. There are two missing values in the data, so we supplemented the data by interpolation.

##### 3.2.2.2 Public health services (PMS)

Currently, the quantification of health and care institutions, beds in health and care institutions, and health technical personnel serve as significant metrics for assessing the quality of public health services (42). Drawing on Yanling and Hua (43) and Wei and Lei (44), this paper constructs the index system for public health services with the number of health and care institutions per 10,000 population, the number of beds in health and care institutions per 1,000 population, and the number of health technical personnel per 1000 population. Among them, health and care institutions mainly include hospitals of all levels and types, primary health care institutions, specialized public health institutions, and other health care institutions. Health technical personnel include various health professionals such as licensed physicians, licensed physician assistants, registered nurses, pharmacists, laboratory technicians, imaging technicians, health inspectors, and trainee physicians. However, it does not include health technicians in managerial positions (e.g., deans, vice-directors). Similar to the previous method of measuring public sports services, the entropy weighting method was used to determine the weights of the indicators for public health services. Finally, the products of the indicator weights and data were summed to obtain public health services in each province.

#### 3.2.3 Control variables

##### 3.2.3.1 Economic development (GDPpc)

Economic development has a complex impact on national health that cannot be ignored (45). This paper uses real GDP per capita to measure the level of economic development and finally takes natural logarithm.

##### 3.2.3.2 Education (Edu)

Health economics holds that education has a significant positive effect on health (46). It is measured in terms of the number of enrolments per 100,000 inhabitants by high education.

##### 3.2.3.3 Environmental pollution (Pollution)

Water and air pollution are main environmental problems in China, seriously jeopardizing national health. Therefore, we use sulfur dioxide emission per unit area, soot and dust emission per unit area, ammonia nitrogen emission per unit area, and chemical oxygen demand per unit area to construct the index system for environmental pollution. The total environmental pollution score was calculated by the entropy method. Finally, it is logarithmized.

##### 3.2.3.4 Urbanization (Urban)

Most literature suggests a negative correlation between adult mortality rates and urbanization (47, 48). Zhu et al. (49) argue that the effects of urbanization on health are heterogeneous by region, age, and sex. It is evident that there is no consensus on the relationship between urbanization and health. Therefore, this paper controls for urbanization, measuring it through the proportion of urban population in total population and logarithmically.

##### 3.2.3.5 Sex ratio (Sex)

Some studies show a correlation between sex ratio and national health (50). For this reason, this paper controls sex ratio and uses the ratio of the number of males to the number of females to measure it.

##### 3.2.3.6 The prevalence of various diseases (Pre)

Infectious diseases are characterized by rapid spread, severe illness, and high fatality rates. This is particularly the case for statutorily reported infectious diseases in Category A and B, which are far more destructive to public health than statutorily reported infectious diseases in Category C. The former is the subject of mandatory and strict management by the Chinese government. To this end, the incidence of statutorily reported infectious diseases in categories A and B is used as a control variable. According to the China Health Statistics Yearbook, statutorily reported infectious diseases in categories A and B include 29 diseases, such as AIDS, tuberculosis, and viral hepatitis. Considering that the yearbook reports the total incidence of statutorily reported infectious diseases in categories A and B at the same time, we adopted the general indicator. Finally, it was logarithmized.

Descriptive statistics of all of the above variables are shown in Table 1.

**Table 1:** Descriptive statistics.

Category	Variable	Observes	Mean	Std. Dev	Min	Max
Dependent variable	MOR	341	0.061	0.008	0.042	0.086
Independent variable	PSS	341	0.127	0.060	0.041	0.397
	PMS	341	0.391	0.103	0.132	0.668
Control variable	GDPpc	341	10.500	0.491	9.210	11.735
	Edu	341	7.781	0.306	6.950	8.766
	Pollution	341	0.078	0.080	0.000	0.562
	SR	341	4.653	0.036	4.562	4.814
	Urban	341	4.034	0.238	3.164	4.506
	Pre	341	5.419	0.356	4.392	6.492

### 3.3 Model design

To verify research hypothesis proposed above, this paper sets up the following baseline regression model (Equation 1):


(1) $$MOR_{\mathrm{it}}=b_{0}+b_{1}PSS_{\mathrm{it}}+b_{2}\mathrm{PM}S_{\mathrm{it}}+b_{3}X_{\mathrm{it}}+b_{0}+\mu_{i}+\omega_{t}+\varepsilon_{\mathrm{it}}$$

Among them, $MOR_{\mathrm{it}}$ is the level of national health of province i in year t, and $PSS_{\mathrm{it}}$ and $\mathrm{PM}S_{\mathrm{it}}$ are, respectively, the level of public sports and health services of province i in year t. X is the set of control variables, including economic development (GDPpc), environmental pollution (Pollution), education (Edu), urbanization (Urban), sex ratio (Sex), and the prevalence of various diseases (Pre). i and t are province and year identifiers. $\mu_{i}$ is the province fixed effect, $\omega_{t}$is the time fixed effect, and $\varepsilon_{\mathrm{it}}$ is a random disturbance term.

## 4 Results

### 4.1 Baseline regression

Table 2 reports the results of baseline regression. Column (1) shows regression results with the province fixed effect, the time fixed effect, and control variables. Columns (2) to (5) are regression results that sequentially add current period, lag one, lag two, and lag three periods of public sports services to column (1). At the same time, column (5) adds public health services.

As shown in columns (2)–(5), regression coefficients for independent variable in the first three columns fail significance test, except for regression coefficient for three-period lag of public sports services, which is significantly negative. Since the independent variable is a negative indicator, this indicates that strengthening public sports services reduces population mortality. The health impacts of public sports programs are characterized by temporal delay and deferred gratification, exhibiting specific enduring attributes. In other words, it is unrealistic to expect health effect of public sports services to be immediately visible, and it will only become apparent after sustained investment and accumulation in public sports services. The finding of this paper is consistent with the welfare cumulative effect of public service policies proposed by Yajing (51). There exists a positive correlation between the quality of public sports services and national health. The first hypothesis of this paper is verified. Our research confirms the cumulative effect of public sports services on the improvement of national health. This can be understood in two ways. First of all, public sports services can achieve positive health improvement effects only through residents’ regular participation in physical activities. Numerous studies on “exercise as medicine” have shown that physical activity has a positive effect on health, but that there are cumulative effects for almost all physical activity (52–54). Secondly, public sports services, like other public services, also have a certain lag effect and cumulative effect on the improvement of national health. Similar to our study, Chunliang et al. (55), through a sample of 281 cities in China from 2003 to 2020, confirmed that the more complete the supply of public services, the faster the accumulation of human capital. That is, public service has a cumulative effect of positive feedback. Therefore, our research consolidates the following viewpoints: Do not expect “instant results.” Government departments should pay attention to the long-term supply of public sports services to better release its cumulative effect.

The regression coefficient for public health services in column (5) is significantly positive, indicating that increasing public health services does not reduce population mortality. The second hypothesis of this paper is tested. However, other similar studies have reached inconsistent conclusions. Evidence suggests that healthcare utilization improves the self-rated health status of older adults in China and the United States (56, 57). However, some of the literature has also found that health care does not have a significant effect on health outcomes (58). All of the above literature has examined only a single health service indicator or a specific population. There is still very little literature that specifically examines the impact of public health services on overall mortality. The striking relationship we have found between public health services and mortality in China must be explained. The possible reasons are as follows: first, evidence from other countries indirectly supports a similar hypothesis. Cochrane et al. (34) found that physician prevalence was significantly and positively associated with mortality among American young people. Second, empirical studies in China have found that public health services improve health by using mortality of perinatal, newborn, and child as inverse indices of health. We argue that population mortality is a more comprehensive and objective indicator than other health indicators about population-specific. Therefore, we speculate that our seemingly amazing research result is of warning and enlightening significance in the special political atmosphere of China. As stated in the previous theoretical analysis, public health services are not directly valid for national health in most countries, and there may be other pathways to improve health (13). Fourth, Filmer and Pritchett (13) argue that there are clear differences between nominal and real public health expenditures in developing countries. In China, the largest developing country, the management of healthcare sector is inefficient (59), and an excessive proportion of medical expenses borne by individual residents delays disease treatment (60). Finally, environmental pollution, which has not yet radically improved, will continue to worsen population mortality, thus increasing burden on healthcare sector.

**Figure 1:**
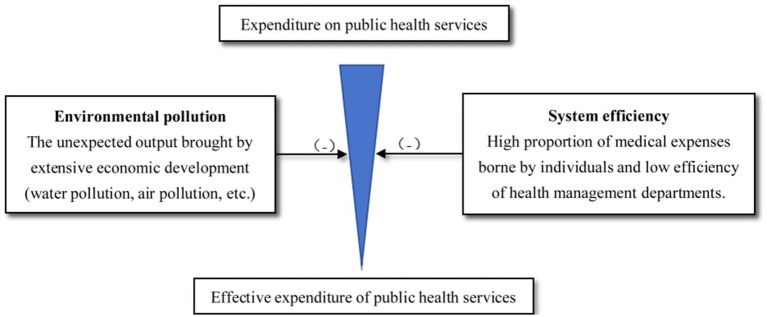
Funnel model of effective expenditure of public health services in China.

On the whole, the increase in the supply of public health services does not necessarily improve national health. Drawing on Filmer and Pritchett (13) complex causal chain from public health services to national health, this paper presents a funnel model of effective expenditure on public health services in China (Figure 1).

As can be seen from Figure 1: (1) Due to the inefficiency of public health service department, the cost of public health service has been increased or the actual expenditure has been reduced; (2) as China is still in a state of economic catch-up for a long time, the desire for higher economic growth and the failure to make an effective green transition have resulted in more unexpected outputs such as water pollution and air pollution, thus increasing the burden of public health services or reducing the actual effective expenditure. Due to the factors including but not limited to the above, the increase in the supply of public health services does not necessarily improve national health.

Among control variables in column (5), regression coefficient of GDP per capita on population mortality was positive but not significant. It can be explained in two ways. First, high population density in economically developed areas provides rapid and easy transmission for infectious diseases, which may increase mortality. Internal mortality factors such as cardiovascular disease (61) and external mortality factors such as traffic accidents (62) are likely to be more damaging to health in economically developed areas. Second, reasons for non-significance may be related to sample, to be revealed by subsequent heterogeneity analyzes. The regression coefficient of environmental pollution is significantly positive, which means that environmental pollution has become a negative factor affecting China’s national health that should not be ignored. It is still very important to continue to combat environmental pollution to promote national health. The regression coefficient of education is negative and significant, consistent with the mainstream view that more years of schooling is beneficial to health. The regression coefficient for urbanization is significantly negative, which may be explained by the fact that health awareness of residents has been increased during urbanization process, which improves their health (63). The regression coefficient for sex ratio is small and do not pass significance test, indicating that gender factor is weakly associated with national health.

### 4.2 Robustness checks

Since public sports services are macro public policies at the national level and it is difficult for national health at the individual level to influence these public policies. Consequently, there is little reverse causality between them. In addition to controlling the double fixed effect of year and province, the following five robustness tests are conducted in this paper to demonstrate robustness of baseline regression results (Table 3).

**Table 2:** Baseline regression.

Variables	(1)	(2)	(3)	(4)	(5)
	MOR	MOR	MOR	MOR	MOR
PSS		0.834 (1.03)	1.062 (1.44)	1.023 (1.37)	1.324* (1.84)
			−0.799 (−1.07)	−0.537 (−0.85)	−0.582 (−0.96)
L2.PSS				−0.741 (−1.34)	−0.195 (−0.44)
L3.PSS					−2.265*** (−4.11)
PMS		2.446** (2.17)	2.471** (2.23)	2.504** (2.28)	2.922*** (2.90)
GDPpc	1.619 (1.29)	0.445 (0.35)	0.438 (0.34)	0.500 (0.38)	0.449 (0.35)
Pollution	1.598** (2.38)	1.343** (2.13)	1.320** (2.07)	1.306* (2.02)	1.323** (2.07)
Edu	−0.666 (−1.21)	−0.922** (−2.14)	−0.944** (−2.26)	−0.930** (−2.25)	−1.030*** (−2.85)
Urban	−2.004 (−1.32)	−2.384* (−1.79)	−2.321* (−1.89)	−2.255* (−1.96)	−1.762* (−1.77)
Sex	−0.014** (−2.22)	−0.011* (−1.85)	−0.011* (−1.89)	−0.011* (−1.78)	−0.009 (−1.51)
Province FE	YES	YES	YES	YES	YES
Year FE	YES	YES	YES	YES	YES
Constant	3.791 (0.35)	18.001 (1.44)	18.046 (1.45)	17.049 (1.35)	16.282 (1.29)
Observations	341	341	341	341	341
R-squared	0.472	0.503	0.505	0.508	0.532
Number of provinces	31	31	31	31	31

***, **, and * indicate significance at the 1, 5, and 10% levels, respectively, with T-values in parentheses; the same below.

#### 4.2.1 Control variables lag one period

Following the approach of Chuanwang et al. (64), the lag terms of all control variables were added to the model to rule out potential endogeneity problems with control variables. There is no significant change in direction and significance in column (1), effectively supporting baseline regression results.

#### 4.2.2 Dependent variable replacement

Although the single maternal mortality rate (MOR1), perinatal mortality rate (MOR2), and mortality of legally reported infectious diseases of categories A and B (MOR3) can hardly reflect the overall health of all populations, we still consider the above three indicators as three substitute variables of national health for the sake of robustness, and the regression results are still robust. Referring to Xueyan et al. (65), the entropy method was applied to calculate weights for three indicators, including maternal mortality rate, perinatal mortality rate, and mortality of legally reported infectious diseases of categories A and B. Then sum them up to obtain proxy variable (MOR4) for national health. The regression result is still valid in column (2), indicating robustness of benchmark regression result.

#### 4.2.3 Winsorize processing

Public sports and health services were subjected to winsorize processing in the [2, 98%] range to avoid possible influence of outliers on the result. The regression coefficients for independent variables remain significantly negative in column (3), showing that regression result is robust.

#### 4.2.4 Shorten time window

Considering the impact of COVID-19 pandemic on mortality in 2020, we shortened time window for regression and conducted empirical studies on the province panel data from 2010–2019. The result, as shown in column (4), shows that regression coefficient of public sports services on mortality remains significantly negative, verifying the robustness of result.

#### 4.2.5 Add a control variable

The incidence of statutorily reported infectious diseases in category A and B does have an impact on the death rate. With the addition of this important control variable, the significance and direction of the regression coefficients of the main explanatory variables remain unchanged and the findings remain robust.

## 5 Further analysis

### 5.1 Interaction between public sports and health services

As mentioned in the previous theoretical analysis, it may not be possible to get a correct conclusion by examining health effect of public health services or public sports services alone. There is a natural property of integration of public sports and health services. For this reason, this section specifically tests whether interaction between public sport and health services has health improvement.

Table 4 presents regression results. Column (1) only includes public sports services and control variables. Column (2) adds public health services to column (1), and regression result echo the previous argument that it does not have significant benefits for national health. Columns (3) and (4) both add the interaction term of public sport and health services to column (2). The former is a mixed regression model, while the latter is two-way fixed effects. The interaction terms in the two different models are significantly negative, and the results are robust. This indicates that public health services have a very positive contribution to health effects of public sports services. The third research hypothesis proposed in this paper was tested.

**Table 3:** Robustness tests.

Variables	(1)	(2)				(3)	(4)	(5)
	Lagged control variables	MOR1	MOR2	MOR3	MOR4	Winsorize [2, 98%]	Shorten time window	Add control variables
L3.PSS	−1.906*** (−3.26)	−4.620*** (−3.46)	−0.908* (−1.97)	−0.942* (−1.72)	−0.279** (−2.58)	−2.246*** (−3.36)	−2.105** (−2.55)	−2.055*** (−4.09)
PMS	3.731*** (4.24)	1.133* (1.69)	1.684*** (3.13)	2.090*** (3.29)	0.194** (2.36)	2.833*** (2.87)	1.619** (2.24)	2.827*** (2.91)
Control	YES	YES	YES	YES	YES	YES	YES	YES
Constant	31.134*** (3.14)	−22.131 (−1.46)	21.749*** (4.59)	0.232 (0.03)	0.799 (1.20)	16.032 (1.29)	10.114*** (7.35)	13.109 (1.00)
Province FE	Yes	Yes	Yes	Yes	Yes	Yes	Yes	Yes
Year FE	Yes	Yes	Yes	Yes	Yes	Yes	Yes	Yes
Observations	341	341	341	341	341	341	310	341
R-squared	0.547	0.771	0.379	0.303	0.732	0.522	0.267	0.551
Number of provinces	31	31	31	31	31	31	31	31

**Figure 2:**
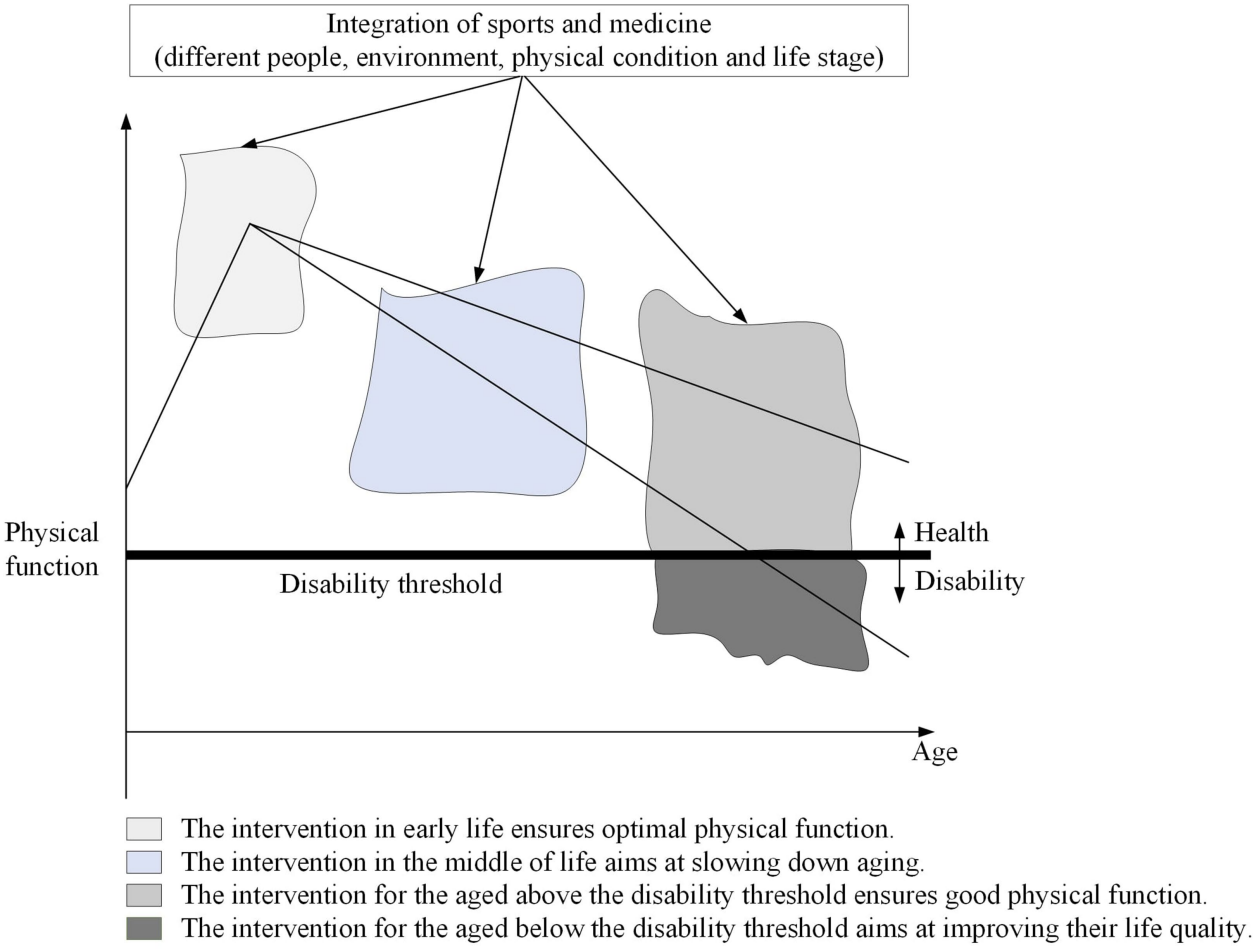
A intervention framework of integration of sports and medicine based on the whole life course.

Compared with the single health effect of public sports service, why is the health improvement effect of public sports service so remarkable once it is combined or interacted with public health service? In our opinion, the theoretical basis behind it is integration of sports and medicine. The interaction between public sports services and public health services belongs to the concept of integration of sports and medicine in essence. For example, the promotion of exercise prescription is the most important content of integration of public health and sports services, which has led to a move forward in the health threshold and a more precise approach to health ownership (25, 66). Although physical activity is an important component of healthy, happy, and independent living throughout the life cycle, this effect can only be amplified when combined with public health services (Figure 2). This also provides richer information for how to dialectically treat “Exercise is Medicine.” Therefore, integration of sports and medicine provides an important theoretical interpretation for health effect of interaction between public health and sports services.

This new discovery provides a wealth of information for integration of sports and medicine. Our study not only confirms the linear relationship between public sports services and national health but also provides new evidence for the complex relationship between integration of sport and medicine and national health. The relationship among sports, medicine, and health is becoming more and more complex in society (67). Integration of sport and medicine is an important element of the “Healthy China” strategy at present, but it is basically at the conceptual level. Although most literature emphasizes health-promoting effect of integration of sports and medicine, they are mostly theoretical explanations, lacking empirical tests (68). Thus, interaction between public sports and health services revealed in this paper has a very positive impact on health and is conducive to understanding of integration of sport and medicine. This has important implications for policy makers and practitioners to be mindful of the coherence between public health and public sports policies, as well as the development of sports medicine as an interdisciplinary discipline (69).

### 5.2 Heterogeneity analysis

The sample of paper covers 31 provinces, autonomous regions, and municipalities in China. It is therefore natural to focus on whether there are significant differences in national health and health effect of public sports and health services across regions. To this end, an existential test was conducted to examine differences in national health across regions. We used China’s population mortality to map differences in national health in 2010 and 2020, respectively.

**Figure 3:**
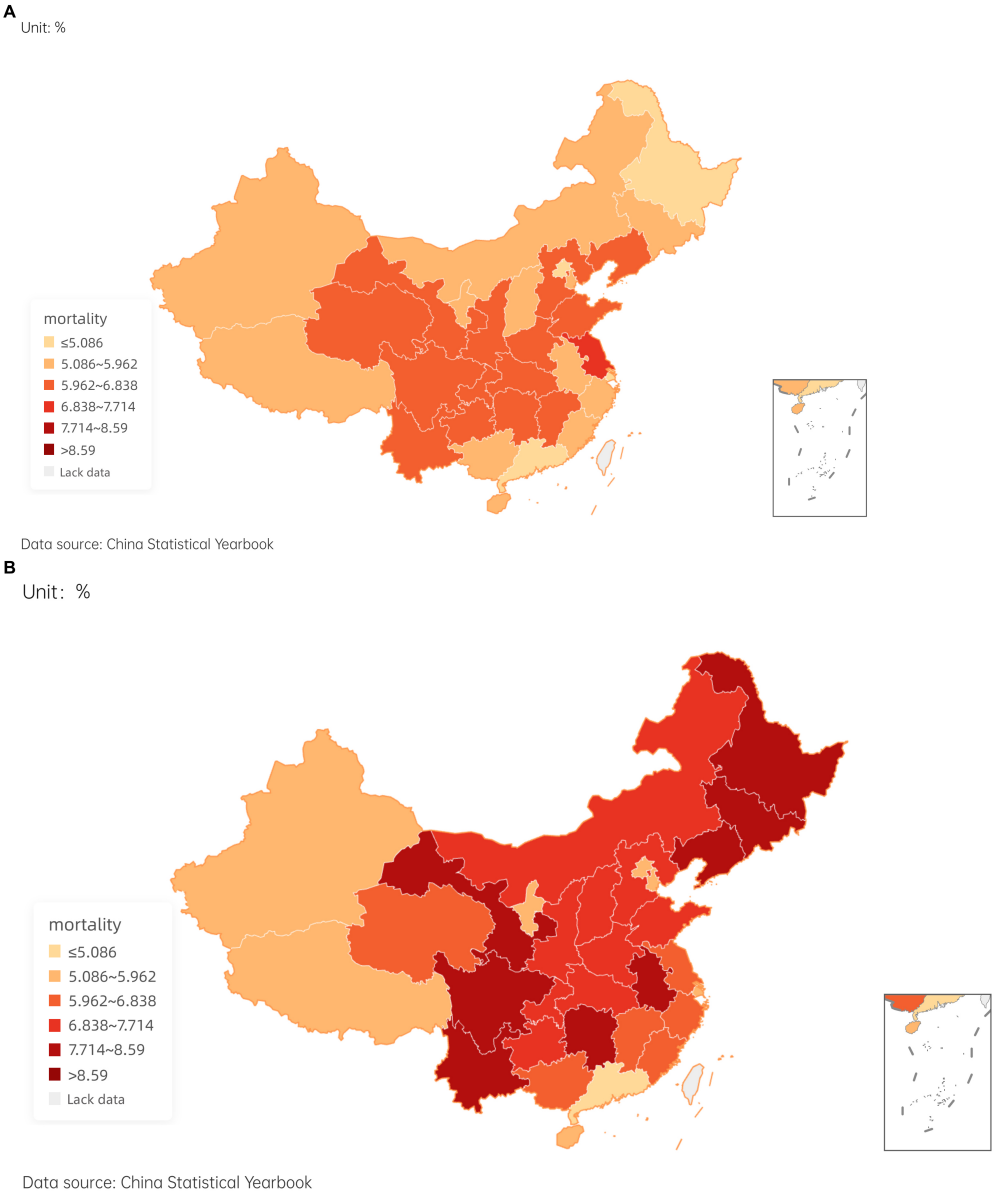
The mortality of provinces in China in 2010 and 2020.

Consistent with our expectations, population mortality of China varies to some extent geographically (Figures 3A,B). Specifically, the high population mortality has experienced a new shift from central to eastern regions. On the whole, western region economically underdeveloped does not necessarily have a higher population mortality rate than central and eastern regions economically developed.

Health inequality is considered a systematic difference among different social groups or regions (70). In my opinion, there are more likely to be deep-seated reasons behind geographical differences in population mortality such as uncoordinated economic development and different climate. Therefore, this paper examines heterogeneity from two distinct viewpoints of regional economic growth and extreme climate, in contrast to existing literature that mostly analyzes heterogeneity through regional distribution.

#### 5.2.1 Heterogeneity of economic development

##### 5.2.1.1 Heterogeneity test for economic development

The Chinese government has placed significant emphasis on the robust advancement of sports and healthcare, with a particular focus on promoting national health. However, an analysis of data extracted from the China Statistical Yearbook 2021 reveals that the allocation of funds towards healthcare and sports constitutes a mere 10.7 percent of the total expenditure in central government general public budgets in 2021. There is a large gap between the proportion of fiscal expenditure on basic public services for sports and healthcare and the requirements of the “Healthy China” strategy. In addition, China exhibits a notable disparity in economic development among its various areas. Therefore, it is worthy of further exploration whether public sports and health services have diverse impacts on national health due to different economic bases. Columns (1) and (2) of Table 5 report the results.

**Table 4:** Interaction term regression.

Variables	(1)	(2)	(3)	(4)
	MOR	MOR	MOR	MOR
L3.PSS	−1.723** (−2.32)	−2.205*** (−3.98)	−5.886*** (−4.77)	−1.034 (−1.47)
PMS		2.739** (2.69)	2.459*** (4.86)	3.140*** (3.58)
L3.PSS × PMS			−11.152** (−2.12)	−7.972*** (−2.82)
Control	YES	YES	YES	YES
Constant	0.472 (0.04)	16.643 (1.31)	10.014*** (5.38)	20.330 (1.58)
Province FE	Yes	Yes	No	Yes
Year FE	Yes	Yes	No	Yes
Observations	341	341	341	341
R-squared	0.488	0.523	0.323	0.533
Number of provinces	31	31	31	31

When public sports services are increased, population mortality is reduced in areas with lower per capita GDP. One possible reason is related to the fact that economically developed regions produce more undesired outputs such as environmental pollution. China’s air pollution is relatively severe in economically developed eastern and central regions (38). We judge that financial expenditure to combat environmental pollution has a certain “crowding out effect” on financial investment of public sports in economically developed areas, thereby diminishing the health benefits associated with public sports services. The finding, to some extent, responds to academic controversy on whether economic growth is beneficial to health (71). Most studies suggest that economic recession is conducive to lower mortality (72–74). A similar conclusion has been obtained from relevant studies in China (75). However, these studies have not paid in-depth attention to the issue of regional variability in health effect of public sports services.

The regression coefficient of public health service is significantly positive in economically developed areas, which may be related to more serious environmental pollution. The regression coefficient of the interaction between public sports services and public health services is as high as-14.309% in economically developed areas, passing the 1% significance test. This outcome should be associated with the advancements achieved in the execution of the “Healthy China” initiative within China. The initiative promotes the deep integration of national fitness and national health to achieve substantial universality of healthy lifestyles by 2030 and a significant reduction in premature mortality from major chronic diseases (76).

It should be noted that most of the regions with low per capita GDP belong to central and western regions. Therefore, this section indirectly supports the research conclusion of previous heterogeneity of regions to some extent.

##### 5.2.1.2 The path analysis of heterogeneity of economic development

It is not a simple promotion or inhibition of economic development on national health, which may be related to the opposite way behind it. First of all, before the inflection point of the Kuznets curve completely across the environment in China, there is a positive relationship between the level of economic development and the non-expected output represented by SO2 and smoke and dust (77). This means that environmental pollution accompanied by economic development has an inhibitory effect on national health. Secondly, the more developed the economy, the higher the fiscal revenue, and it is more likely to have sufficient economic foundation and material conditions to improve the supply of public sports services, thereby indirectly promoting national health (78). Therefore, we predict that the differences in environmental pollution and fiscal revenue are the substantive reasons for differences in health effects of public sports services under different levels of economic development. To investigate the reasons for differences, we used the interaction terms of environmental pollution and fiscal revenue with public sports services (PPS), public health services (PMS), and their interaction terms (PPS × PMS) respectively. The regression equation is shown in Eqs. (2, 3). In order to save space, we only reported the coefficients and significance of the interaction items in the Table 6.


(2) $$\begin{array}{l} MOR_{\mathrm{it}}=\alpha_{1}\times PSS_{\mathrm{it}}+\alpha_{2}\times \mathrm{PM}S_{\mathrm{it}}+\alpha_{3}\\ \;\times \left(PSS_{\mathrm{it}}\times \mathrm{PM}S_{\mathrm{it}}\times \mathrm{Pollutio}n_{\mathrm{it}}\right)+\alpha_{4}\times X_{\mathrm{it}}+\varepsilon_{\mathrm{it}} \end{array}$$

(3) $$\begin{array}{l} MOR_{\mathrm{it}}=\beta_{1}\times PSS_{\mathrm{it}}+\beta_{2}\times \mathrm{PM}S_{\mathrm{it}}+\beta_{3}\\ \times \left(PSS_{\mathrm{it}}\times \mathrm{PM}S_{\mathrm{it}}\times FI_{\mathrm{it}}\right)+\beta_{4}\times X_{\mathrm{it}}+\varepsilon_{\mathrm{it}}\; \end{array}$$

**Table 5:** Test results of environmental pollution and fiscal revenue.

	(1) MOR	(2) MOR
L3.PSS × Pollution	0.400* (1.95)	
PMS × Pollution	0.214 (0.59)	
L3.PSS × PMS × Pollution	0.536** (2.19)	
L3.PSS × FI		−1.755* (−1.97)
PMS × FI		−1.502*** (−3.26)
L3.PSS × PMS × FI		−0.995*** (−3.36)
N	341	341
R2	0.517	0.529

$FI_{\mathrm{it}}$ is the per capita revenue in local government general public budgets of province i in year t.

The regression results in Table 6 show that environmental pollution inhibits the health effects of public sports services and the interaction of public sports and health services (integration of sports and medicine), respectively. On the contrary, local fiscal revenue significantly promoted their health effects. In other words, public sports services have a more significant improvement in national health in areas where environmental pollution is better controlled and public financial strength is stronger. Although it is open to question only using environmental pollution and local fiscal revenue as channel variables of economic development, the above regression results are in line with our expectations. It is confirmed that the differential impact of economic development on national health is related to the internal mechanism with opposite action directions behind it.

It should be noted that environmental pollution aggravated the inhibitory effect of public health service (PMS) on national health, although this effect did not pass the significance test. This result is in line with expectations, indicating that environmental pollution is indeed an unexpected output of economic development, which is unfavorable to national health. Local fiscal revenue has greatly alleviated the inhibitory effect of public health services (PMS) on national health. This result is also in line with expectations, reaffirming that economic development is an important material guarantee for national health and welfare.

#### 5.2.2 Heterogeneity of extreme temperature

The relationship between sports and climate is well established in academia (79, 80). The latest research literature from the authoritative medical journal “The Lancet” and the most authoritative journal in China “Chinese Science Bulletin” both believe that extreme temperature is an important external influence on population health and rapidly changing temperature endangers human health and increases the risk of death (81–83). This provides a basis for the heterogeneity grouping test based on extreme temperatures. Therefore, it is necessary to explore whether public sports and health services have differential impacts on national health under extreme weather.

Drawing on Qianni, Chuan (84), this paper uses the number of extremely high temperatures and extremely low temperatures as grouping variables. Columns (3)–(6) of Table 5 report grouping results for extreme temperatures. When public sports services are increased, population mortality is significantly lower in areas with fewer extremely high temperatures and extremely low temperatures. Public health services are still positively associated with mortality. Regression coefficients of interaction between public sports and health services are still as high as-10.521% and-9.911%, respectively, and pass the significance test, once again robustly validating the second and third research hypotheses. As extreme temperatures trigger premature deaths in vulnerable groups such as patients, this increases pressure on public sports and health services in areas severely affected by extreme temperatures. The results suggest that strengthening integration of sports and medicine is an effective response to extreme climate. Therefore, policymakers in the healthcare and sports sectors need to take synergistic action to address climate change (85).

**Table 6:** Heterogeneity tests.

Variables	(1)	(2)	(3)	(4)	(5)	(6)
	High GDP	Low GDP	More extremely high temperatures	Less extremely high temperatures	More extremely low temperatures	Less extremely low temperatures
L3.PSS	−0.423 (−0.64)	−2.822** (−2.14)	−1.029 (−1.42)	−2.231** (−2.16)	1.798 (1.38)	−1.844** (−2.50)
PMS	4.131*** (2.91)	2.123 (1.30)	1.885 (1.49)	3.665*** (3.70)	5.517*** (3.78)	1.687** (2.64)
L3.PSS × PMS	−14.309*** (−3.02)	−6.953 (−1.40)	−7.580 (−1.55)	−10.521** (−2.73)	−17.629 (−0.86)	−9.911*** (−2.96)
Control	YES	YES	YES	YES	YES	YES
Constant	3.845 (0.41)	5.475 (1.09)	−26.471** (−2.81)	15.604 (1.07)	49.556*** (3.54)	−14.869* (−1.79)
Province FE	Yes	Yes	Yes	Yes	Yes	Yes
Year FE	Yes	Yes	Yes	Yes	Yes	Yes
Observations	169	172	137	204	95	246
R-squared	0.527	0.327	0.612	0.537	0.772	0.447
Number of provinces	25	22	15	23	10	23

## 6 Research conclusions and policy implications

### 6.1 Research conclusions

There has been a rebound in population mortality in the last 10 years. At the same time, investment in public sports and health services continues to increase. This abnormal problem needs to be discussed specifically and clearly. In this paper, public sports and health services are both included in the benchmark analysis framework. Based on the panel data of 31 provinces in China from 2010 to 2020, this paper estimates different effects of public sports services, public health services, and their interaction on population mortality by using the two-way fixed effect model.

The main conclusions of this paper are as follows: (1) increasing the supply of public sports services is conducive to reducing population mortality. However, health effect of public sports services is characterized by delayed realization. That is to say, its health effects can only be realized through sustained investment and accumulation over time. (2) The integration of sports and medicine has a strong health effect, and simply increasing public health services is not beneficial to national health. This sheds new light on the understanding of “fundamental” and “incidental” relationships between public sports and health services and integration of sports and medicine. (3) The health effect of interaction between public sports and health services is heterogeneous in terms of economic development and extreme temperatures. When economy is developed or extreme temperature is less, this effect is more prominent.

### 6.2 Policy implications

This paper has the following important policy implications:

#### 6.2.1 Focus on a higher level of public sports services

Our research suggests that when public sports services increased, population mortality decreased significantly. To this end, it is imperative to actively promote the “a Leading Sporting Nation “strategy and the “Fitness for All” plan. In the future, an important policy trend will involve enhancing public sports services through initiatives such as bolstering the personalized sports prescription repository and advancing social sports instructor teams.

#### 6.2.2 Attach great importance to the role of integration of sports and medicine for health

The research suggests that public health services exert health effects by interacting with public sports services. Therefore, there is still a need to correctly address the relationship between “treatment” and “prevention” and between “active health” and “passive health” given the current national strategy of “healthy China,.” This strategy underscores the pivotal significance of integrating sports and medicine within the context of national health. It advocates for a proactive approach that addresses the underlying causes of health issues rather than solely focusing on symptom management. By doing so, it aims to mitigate excessive dependence on public health services.

#### 6.2.3 Alleviate the inhibitory effect of extreme temperatures on health effects

China’s growing population and greenhouse gas emissions enhance the threat of severe temperatures on population health (86), undermining public sports services’ positive effects on national health and burdening the healthcare system. For this reason, the sports and healthcare sectors should play a more important role and work together to fight against extreme climate. As far as we know, China has not specifically promulgated a national-level policy on climate adaptation and health at present. Therefore, there is a need to specifically launch a public services adaptation plan to cope with the health risks of extreme climate and to introduce the most appropriate interventions for public sport to improve national resilience to extreme climate.

### 6.3 Limitation

There is some room for extended research in this paper. First, in terms of data availability, the analysis in this paper is limited to provincial panel data for 2010–2020. Therefore, future research could be extended to the city level and time forward, which in turn may be able to yield additional and fuller insights. Second, we find that the interaction between public sports services and public health services has different health effects due to differences in economic development levels and extreme climate. An in-depth analysis of reasoning has not been conducted as a result of the limited quantity of papers available. Future research can follow this direction to focus on the mechanism of climate or economic development level on the health effects of public sports services and/or public health services. We believe that this is a direction with research value.

## Data availability statement

The original contributions presented in the study are included in the article/supplementary material, further inquiries can be directed to the corresponding authors.

## Author contributions

LC: Conceptualization, Formal analysis, Writing – original draft, Writing – review & editing. JC: Formal analysis, Writing – original draft, Writing – review & editing. YG: Writing – review & editing. QB: Writing – review & editing, Methodology. JH: Writing – review & editing, Conceptualization. NT: Writing – review & editing.
